# Data for differentially expressed microRNAs in saturated fatty acid palmitate-treated HepG2 cells

**DOI:** 10.1016/j.dib.2016.11.062

**Published:** 2016-11-19

**Authors:** Won-Mo Yang, Kyung-Ho Min, Wan Lee

**Affiliations:** a Department of Biochemistry, Dongguk University College of Medicine, Gyeongju 780-714, South Korea; b Endocrine Channelopathy, Channelopathy Research Center, Dongguk University College of Medicine, Goyang, 410-773, South Korea

**Keywords:** MicroRNAs, Palmitate, Saturated fatty acids, Obesity, Hepatocyte

## Abstract

Certain microRNAs (miRNAs) targeting the molecules in the insulin signaling cascades are dysregulated by saturated fatty acids (SFA), which can lead to insulin resistance and type 2 diabetes. This article reports the accompanying data collected using miRNAs microarrays to identify the changes in miRNA expression in HepG2 cells treated with SFA palmitate. Differentially expressed miRNA analyses in HepG2 cells showed that a range of upregulated (>1.5-fold) or downregulated (<0.5-fold) miRNAs. Further extensive insights into the implications of miRNAs, particularly miR-1271, in HepG2 cells can be found in "MiR-1271 upregulated by saturated fatty acid palmitate provokes impaired insulin signaling by repressing INSR and IRS-1 expression in HepG2 cells" (W.M. Yang, K.H. Min, W. Lee, 2016) [1].

**Specifications Table**

**Table:** 

Subject area	*Biology, Biochemistry*
More specific subject area	*Obesity, Metabolism, MicroRNA*
Type of data	*Excel files*
How data was acquired	*Affymetrix GeneChip microarray analyses of miRNAs*
Data format	*Analyzed*
Experimental factors	*Palmitate treatment, RNA Isolation, Affymetrix Genechip miRNA microarray*
Experimental features	*Differentially expressed miRNAs of the HepG2 cells treated with SFA palmitate were analyzed using Affymetrix GeneChip miRNA microarray.*
Data source location	*Dongguk University School of Medicine, Gyeongju 780-714, Korea*
Data accessibility	*The data are available with this article*

**Value of the data**

• The data highlight the biological significance of the miRNAs involved in the pathogenesis of SFA-induced metabolic diseases.
• These results can be compared with gene expression analysis from other cell or tissue types in obesity.
• The differentially expressed miRNAs in this dataset could be applied in further functional studies of the cellular and systemic phenotype changes resulting from SFA-induced obesity and metabolic diseases.

## 1 Data

The high dietary intake of saturated fatty acids (SFA), which is the leading cause of obesity, frequently causes ectopic lipid accumulation and increase the risk of insulin resistance in non-adipose tissues, such as the liver and skeletal muscle [2]. The expression of certain miRNAs targeting the insulin signaling molecules is modulated aberrantly in diet-induced obesity, which participates actively in the pathogenesis of insulin resistance [3], [4]. A previous study reported that SFA palmitate induces miR-1271 in HepG2 hepatocytes, and the expression of INSR and IRS-1 is suppressed by targeting their 3’UTR directly [1]. This means that certain miRNA induced by SFA could be linked causally to the development of hepatic insulin resistance and further to type 2 diabetes. This paper reports accompanying data collected from Affymetrix GeneChip microarrays to identify the changes in miRNA expression in HepG2 cells treated with SFA palmitate. Differentially expressed microRNA analyses in HepG2 cells (Supplementary File. 1) revealed a range of miRNAs upregulated more than 1.5-fold (Supplementary File. 2) or downregulated less than 0.5-fold (Supplementary File. 3). Among those differentially expressed miRNAs, upregulated miRNAs have implications on the reduction of INSR and IRS-1 observed in palmitate-treated HepG2 cells [1]. Further analysis of the data and insights into the implications of miRNAs, especially miR-1271, in HepG2 cells are reported in another publication [1].

## 2 Experimental design, materials and methods

### 2.1 Cells and palmitate treatment

HepG2, a human liver cancer cell line, was purchased from ATCC (#77400). The HepG2 cells were grown in MEMα supplemented with 10% FBS and 1% penicillin-streptomycin (Gibco) in an atmosphere containing 5% CO_2_ at 37 °C. The cells from passages 3–10 were used for the following experiments. A fatty acid-free bovine serum albumin (BSA, Bovogen, VIC, Australia)-conjugated palmitate (Sigma-Aldrich) solution was prepared, as described previously [5]. Briefly, BSA and sodium palmitate were dissolved completely in 150 mM NaCl by heating at 37 °C and 70 °C, respectively. The BSA solution was added dropwise to the palmitate solution at 37 °C with continuous stirring until the palmitate to BSA molar ratio was 6:1. The BSA-conjugated palmitate and BSA vehicle was aliquoted and stored at −80 °C. The HepG2 cells were seeded at a density of 5×10^5^/well in a six-well plate. On the next day, the cells were treated with BSA-conjugated palmitate (0.5 mM) for 0–18 h. The control cells were treated with the BSA vehicle. Where applicable, the cells were treated with or without 100 nM insulin during the final 30 min of incubation.

### 2.2 RNA extraction and quality check

The total RNA from the HepG2 cells was extracted using a miRNeasy Mini Kit (Qiagen) according to the manufacturer׳s instructions. The purity and integrity of the RNA were assessed using a ND-1000 Spectrophotometer (NanoDrop) and Agilent 2100 Bioanalyzer (Agilent Technologies). Equal amounts of RNA from five mice were pooled together and used for the microarray.

### 2.3 miRNA arrays analysis

The total RNA described above was prepared and subjected to an Affymetrix Genechip miRNA 4.0 array (Affymetrix, Santa Clara, CA, USA) process according to the Affymetrix technical instructions. Briefly, 600 ng RNA was labeled with a FlashTag™ Biotin RNA Labeling Kit (Genisphere, Hatfield, PA, USA). The labeled RNA was quantified, fractionated, and hybridized to the miRNA microarray according to the manufacturer׳s instructions. The labeled RNA was heated to 99 °C for 5 min and incubated at 45 °C for 5 min. RNA-array hybridization was conducted with agitation at 60 rpm for 16 h at 48 °C on an Affymetrix^®^ 450 Fluidics Station. The chips were stained on a Genechip Fluidics Station 450 (Affymetrix), and scanned using an Affymetrix GCS 3000 scanner (Affymetrix). All signals were normalized according to the quantile method after a log 2 transformation to make them comparable across microarrays.

## Appendix A Supplementary material

Supplementary material Supplementary File 1. Raw data of Affymetrix GeneChip miRNA analysis (Excel file: Raw data.xlsx). Supplementary File 2. Palmitate - upregulated miRNAs (Excel file: HFD - upregulated miRNAs.xlsx). Supplementary File 3. Palmitate - downregulated miRNAs (Excel file: HFD - downregulated miRNAs.xlsx).
